# Measuring irritability across childhood, adolescence, and young adulthood: an investigation of measurement invariance by age, sex, and informant

**DOI:** 10.1007/s00787-025-02817-3

**Published:** 2025-07-12

**Authors:** Aikaterini Bekiropoulou, Olga Eyre, Jon Heron, Anita Thapar, Lucy Riglin

**Affiliations:** 1 Wolfson Centre for Young People’s Mental Health and Centre for Neuropsychiatric Genetics and Genomics, Division of Psychological Medicine and Clinical Neurosciences, Cardiff University, Hadyn Ellis Building, Maindy Road, Cardiff, CF24 4HQ UK; 2 Population Health Sciences and MRC Integrative Epidemiology Unit, University of Bristol, Bristol, UK

**Keywords:** DAWBA, ARI, ALSPAC, Irritability, Measurement invariance

## Abstract

Irritability is a transdiagnostic phenomenon, frequently present in youth mental health services, and associated with multiple adverse outcomes. Irritability research suggests developmental, sex, and informant differences, but robust assessment requires comparison of the same or equivalent measures. Studies on irritability measurement invariance (equivalence) across age, sex, and informant remain limited. This research investigated measurement invariance across age, sex, and informant (parent/self), in the Avon Longitudinal Study of Parents and Children. Primary analyses assessed age (*N* = 9,809) and sex (*N* = 9,803) invariance in irritability measured using the parent-rated Development And Well-Being Assessment (DAWBA) at ages 7–25, and informant invariance (parent- versus self-report) was tested at age 25, separately for males (*N* = 2,494) and females (*N* = 3,408). We did not find evidence of even weak (metric) invariance across age, indicating that the DAWBA-measured irritability may not be developmentally comparable. Findings by sex were mixed, with strict invariance suggested at age 13, strong at age 7, weak at age 25, and not even weak at ages 10 and 15, suggesting that the DAWBA may not always capture the same irritability construct across sex. Informant invariance levels were measure- specific. The DAWBA demonstrated strict invariance across informants for both males and females, suggesting equivalent interpretation of irritability items and similar residuals at age 25 for self- and parent-reports. However secondary informant analyses, assessed separately for males (*N* = 2,505) and females (*N* = 3,415) in the Affective Reactivity Index (ARI) at age 25, did not support even weak invariance for males, but found evidence of strict invariance for females. Our findings suggest that for the DAWBA, comparisons of mean irritability scores are likely valid across parent-/self-reports at age 25, but incomparable across age and sex; for the ARI, parent-/self-reports may be comparable at age 25 for females, but not males.

**Supplementary Information:**

The online version contains supplementary material available at 10.1007/s00787-025-02817-3.

## Introduction

Severe irritability, broadly defined as an elevated disposition towards anger and provocation relative to peers [1], represents a frequent reason for youth mental health evaluation and intervention [2, 3]. Epidemiological evidence suggests that clinically-significant irritability affects around 0.12-5% of children and adolescents in community samples [4] while prevalence in clinical cohorts can be higher [5]. Notably, irritability is integral to 15 DSM-5 diagnoses, often co-occurring with conditions like attention deficit hyperactivity disorder, autism spectrum disorder, anxiety, and depression [6]. Irritability is influenced by complex genetic predispositions [2] and environmental adversities [7], and typically manifests through recurrent temper outbursts, chronic frustration, and sustained periods of irritable mood. Furthermore, contemporary reviews have linked persistent irritability to long-term consequences across various psychosocial functioning domains, including increased susceptibility to anxiety, depression, high-risk behaviours, and suicidality [8], while associations with adverse financial, educational, and social outcomes have been suggested too [9, 10]. Considering the transdiagnostic nature and multifaceted consequences irritability entails, employing reliable assessment measures, is essential.

During childhood, irritability is commonly evaluated through parental reports, whereas in adulthood, self-reports are often utilised [1]. Although multi-informant studies (e.g., [11–13]), hold some promise, rater-agreement is typically modest, and the extent to which different raters report on the same construct, remains unclear [8]. Developmental research spanning childhood-young adulthood is necessary to examine changes across age; however, such robust examination requires employing the same or equivalent measure at each assessment [14]. Evidence on whether irritability measures capture the same latent construct across age, sex, and informant, is currently limited, and it is therefore unclear whether differences in irritability across these areas reflect genuine differences. One way of investigating this involves testing measurement invariance (i.e., the consistency of measurement across different groups [15]). Our study therefore aimed to assess three increasingly-stringent measurement invariance levels (configural, metric/weak, scalar/strong; Table 1, Supplementary Fig. 1) in irritability measures across age, sex, and informant.

**Table 1:** Definitions of measurement invariance levels for ordinal items

Invariance Level	Definition	Invariance Interpretation	Non-Invariance Interpretation
Configural	Similar factor structure.	The same items of an assessment tool tap onto the same factor (construct) across groups.	Individual items may vary in their representation of the same factor/construct across groups.
Weak/Metric	Similar factor loadings.	Individual items of an assessment tool are interpreted similarly and measure the same factor with equal strength and direction across groups.	Individual items may measure the same factor with varying strength and direction across groups.
Strong/Scalar	Similar factor loadings and thresholds.	Differences in latent factor means represent true differences in the level of the construct across groups.	Differences in latent factor means may not reflect genuine differences of the construct across groups.
Strict/Residual	Similar residual variances (sum of item-specific variance and error variance).	Differences in individual item scores reflect true differences in the level of the construct across groups	Differences in individual items scores may represent group-specific variance and not true differences in the level of the construct

### Measuring irritability across age, sex, and informant

Even when the same irritability measure is used at multiple ages, the degree to which the same items tap onto similar levels of irritability is unclear, because normative levels of irritability can vary developmentally. For instance, temper tantrums, frequently used in irritability assessment, are common childhood presentations, but typically diminish through adolescence [2, 16], attributed to cognitive maturation of self-regulation processes [17, 18], social skills acquisition [19], and language development [20]. Therefore, the intensity and frequency of temper tantrums and, consequently, the extent to which they index severe irritability, likely varies with age; endorsing temper tantrum-related items in adolescence or adulthood may suggest higher irritability severity compared to childhood. Indeed, work examining irritability measurement invariance as part of an irritable/defiance dimension, has found partial strong invariance (i.e., weak/strong across different items) across ages 12–25 [21]. Simultaneously, research specifically investigating irritability with the Affective Reactivity Index (ARI) supported strong invariance across adolescence [22], although it is unclear if this would extend to younger or older ages.

Most irritability research explores childhood and adolescence (e.g., [2, 4, 23]), leaving uncertainty on whether adult irritability experience is accurately captured in measurement tools commonly employed in child psychiatry research. For instance, research in adult outpatients suggests that adult irritability typically exhibits stronger mood than behavioural components, compared to younger cohorts [24]; hence, emotional symptoms (e.g., being touchy or easily annoyed) may be endorsed more frequently than behavioural ones (e.g., tantrums) in adults. Therefore, when measuring irritability developmentally, different items may capture differing levels of irritability severity at different ages, suggesting that measures may lack strong age invariance.

Research also indicates possible measurement non-invariance in irritability by sex. Gender norms and systemic differences in societal expectations may influence the broader expression of psychopathology [25] and therefore, the development and interpretation of psychometric scales. Indeed, sex differences in irritability-reporting have been observed, whereby men were more likely to interpret symptoms like “grouchy” and “looking for trouble” as indicative of irritability, while women more prone to attribute presentations like “intolerant” and “moody” to irritability [26]. Simultaneously, externalising symptoms may not always be considered socially appropriate for females to display [27], hence girls’ presentations could be less overt and internalised in nature. Indeed, research supports that female irritability may manifest as more emotional/mood-like, especially in adolescence, whereas it tends to be more behavioural in boys [28]. This indicates that the same items may not be uniformally indicative of irritability across sexes, and thus, irritability may not meet criteria for even weak sex invariance.

Finally, research suggests that irritability may be interpreted differently according to the informant (i.e., parent/self). A recent study of clinical and community samples spanning childhood-adulthood [29] found that the same irritability items, measured with the ARI, indicated differing severity levels in parent and self-reports (weak, but not strong invariance). Furthermore, longitudinal research using various psychometric tools also suggests that moderate irritability severity may not be accurately captured across both parental and self-reports [23]. Moreover, informant disparities may be larger for emotional/mood-related irritability aspects, which parents may not observe directly, and young people could be less likely to disclose [30]. This suggests that emotional symptoms may need to be more severe for external observers to notice. Indeed, research suggests relatively weak inter-rater consensus in affective symptomatology [31, 32]. Hence, informant differences in interpreting irritability items may be particularly notable in girls, if female irritability is more emotional/mood-like than male; this may indicate lack of even weak invariance by rater, specifically for females.

### The current study

This study aimed to evaluate irritability measurement across childhood, adolescence, and young adulthood, by examining measurement invariance across age, sex, and informant. We investigated three stringent levels of measurement invariance (configural, weak/metric and strong/scalar) in a large UK-based longitudinal population cohort. Specifically, we examined invariance by age and sex in the parent-reported Development and Well-Being Assessment (DAWBA) [33] across ages 7–25, and by rater (parent/self) stratified by sex at age 25. In secondary analyses, we explored informant invariance stratified by sex at age 25 in the ARI [11].

We had three hypotheses:

#### Hypothesis 1

Irritability would demonstrate weak (metric) measurement invariance by age: all irritability items would load onto one irritability factor similarly across age, but item endorsement would reflect different severity levels at different ages (i.e. not strong invariance).

#### Hypothesis 2

All irritability items will load onto one irritability factor for both males and females (configural invariance) but would not meet criteria for weak measurement invariance by sex: the degree to which individual items would load onto one irritability factor would differ by sex.

#### Hypothesis 3

A: For males (age 25), irritability would show weak but not strong measurement invariance by informant: all items would load onto one irritability factor similarly across raters (weak invariance), but item endorsement would show different severity levels by informant (not strong invariance).

B: For females (age 25), all irritability items will load onto one irritability factor across raters (configural invariance), but would not meet criteria for weak invariance: the extent that individual items would load onto one irritability factor will differ by informant.

## Method

The study was pre-registered through the Open Science Framework (OSF) after data collection but prior to data access and analysis (https://osf.io/5gb64/). We had not planned to extend our investigation to residual invariance testing; however, on finding evidence of scalar invariance for some of the models, we continued testing for this more stringent form of invariance. We had also intended to conduct analyses using the automated measurement invariance function in Mplus; however, this would not have enabled us to examine metric and residual invariance for age and sex. We therefore ran our models manually, using the same model constraints as the automated models for configural and scalar invariance.

### Sample

#### The Avon longitudinal study of parents and children (ALSPAC)

The Avon Longitudinal Study of Parents and Children (ALSPAC) is a well-established UK prospective longitudinal birth cohort. Pregnant women residing in Avon, UK, with expected delivery dates between 01/04/1991-31/12/1992, were invited to participate. Initially, 14,541 pregnancies (14,203 unique mothers) were enrolled; 13,988 children were alive one year postpartum. Follow-up recruitment occurred when children reached approximately 7 years, resulting in 15,447 total enrolled-pregnancies (14,833 unique mothers; 14,901 children alive one year postpartum). Participants’ data have been collected at multiple ages, through various methods (e.g., questionnaires, in-person clinic assessments) and sources (e.g., parent-/self-reports). Study data were collected and managed using REDCap electronic data capture tools hosted at the University of Bristol. REDCap (Research Electronic Data Capture) is a secure, web-based software platform designed to support data capture for research studies [34]. More information on ALSPAC, including assessment waves, can be found on other sources [35–37]; data details are available through the fully-searchable data dictionary and variable search tool (https://www.bristol.ac.uk/alspac/researchers/our-data/). In twin pregnancies, we included the first-born child, with at least one irritability measure. Demographic information is presented in Supplementary Table 1.

### Measures

#### Irritability

In keeping with previous research (e.g., [38]), irritability was primarily assessed using the Development And Well-Being Assessment (DAWBA) [33], an extensively validated [39–41] research diagnostic interview, assessing various youth mental health disorders. Parent-reported data were utilised across five age-points (approximately 7, 10, 13, 15 and 25 years), using three Oppositional Defiant Disorder (ODD) irritability items (“severe temper tantrums”, “touchy and easily annoyed”, “angry and resentful”), reflecting the past six months. These items were rated on a 3-point scale (“no more than others”, “a little more than others”, “a lot more than others”). At age 25 years the DAWBA was additionally completed by the young people themselves.

Data from the parent- and self-rated Affective Reactivity Index (ARI) [11] (age 25) were examined as secondary analyses. The ARI is a 7-item scale specifically designed to measure irritability via parental- and self-reports, on a 3-point scale (“not true”, “somewhat true”, “certainly true”). It assesses the frequency, threshold and duration of irritable mood (“easily annoyed by others”, “often loses temper”, “stays angry for a long time”, “angry most of the time”, “gets angry frequently”, “loses temper easily”) and functional impairment (“overall irritability causes problems”), over the past six months; the impairment item was excluded from our analyses. Notably, ARI has been validated in diverse childhood- and adult-samples and possesses excellent internal consistency and test-retest reliability for clinical and non-clinical populations [11, 42, 43]. A summary of DAWBA-ODD irritability items/ARI data categories is displayed in Supplementary Table 2.

### Analyses

#### Statistical software

Data management and descriptive analyses were conducted in R (4.3.3) [44] and main analyses in Mplus (8.10) [45].

#### Statistical analyses

We used Confirmatory Factor Analysis (CFA), to investigate four increasingly-constrained measurement invariance levels: configural, metric/weak, scalar/strong and residual/strict [46, 47]. This process tested if the fundamental factorial pattern of irritability (configural invariance), the relationships between the individual items and the irritability factor (metric invariance), the item endorsement (scalar invariance) and the item-specific and error variance (residual invariance) are similar across age, sex and informant. More information on measurement invariance is presented in Table 1 and visualised in Supplementary Fig. 1. Following computational demands associated with using Maximum Likelihood-Robust (MLR) estimator, according to our pre-registered protocol, we estimated our models with Weighted Least Squares Mean and Variance adjusted (WLSMV) estimation. WLSMV determines each covariance independently of other model’s data and is specifically designed for ordinal categorical data; it has been found to provide less biased estimates than other estimators, especially in large samples [48], while its efficiency in parameter estimation has been supported in models of both lower (e.g., our three-indicator DAWBA-ODD irritability), and higher complexity (e.g. our six-indicator ARI models), assuming large samples [46, 49]. Furthermore, we used Delta parameterization (Mplus’ default) for the configural, metric and scalar models; Theta parameterization was used for the residual model, as this allows investigation of residual variances [45, 47]. Our items were analysed as categorical, following recommendations suggesting that indicators of 3 ordinal-response categories are treated as categorical [49]. Missingness was addressed with WLSMV’s default, i.e., pairwise deletion, which maximises sample sizes while limiting missing data biases [45]. Details of how parameters were fixed/freed for each invariance level and model are in Supplementary Tables 3 and 4.

While some scholars support testing varying loadings (metric invariance) and thresholds (scalar invariance) together, we followed Bowen’s & Masa’s [50] recommendations, testing these in a 4-step approach, separately; this method allows clearer observations and interpretations of non-invariance sources, as loadings and thresholds contribute different information, while it reduces the number of non-invariant parameters being modelled. To identify our baseline model, we fit a configural invariance model, evaluated using the approximate fit indices (AFI) i.e., Comparative Fit Index (CFI), Root Mean Square Error of Approximation (RMSEA) and Standardized Root Mean Squared Residual (SRMR), with acceptable values: ≥0.95, ≤ 0.06 and ≤ 0.08, respectively [51]. Model fit for the nested metric, scalar and residual models was also assessed by chi-square difference testing, conducted using the DIFFTEST option in Mplus, a variant of the standard method which is appropriate for the WLSMV estimator [45]. As this approach may be oversensitive to minor misspecifications in large sample sizes [52], and because of the large number of planned tests (total 30: see details in Supplementary Materials), we employed Bonferroni-corrected p-value (*p* = 0.05/30 = 0.0017) [53]. Metric, scalar and residual invariance were also evaluated using the AFI, which have been found less influenced by sample sizes [52]. Therefore, metric invariance was supported when ΔCFI<-0.01, ΔRMSEA < 0.015, ΔSRMR < 0.03 and DIFFTEST chi-square *p* > 0.0017, while scalar and residual invariance when ΔCFI<-0.01, ΔRMSEA < 0.015, ΔSRMR < 0.01 and chi-square *p* > 0.0017 [54]; these AFI cutoffs have been found to adequately identify model fit in large sample sizes (≥ 1000) with ordered-categorical predictors [55].

In the absence of strong theoretical rationale guiding our selection of reference indicators a priori, the first item of each scale was initially set as the reference indicator. Recognising that a non-invariant reference indicator may result in biased models [56], we conducted sensitivity analyses using the second item as reference indicator. Further, where we found evidence of non-invariance, post-hoc analyses were conducted to investigate partial (item-level) invariance: details are provided in the Supplementary Materials and findings in the Supplementary Tables 8 and 9.

## Results

Irritability prevalence across age, sex, and informant is visualised in Supplementary Figs. 2–5.

### DAWBA-ODD irritability items

#### Hypothesis 1: invariance across age

To explore measurement invariance across age, we first assessed a baseline (configural) model, fitting a single latent factor for parent-rated DAWBA-ODD irritability items (7–25 years), with free loadings and thresholds across age. This provided acceptable fit (Table 2; Model 1.1); therefore, we tested metric invariance (Table 2; Model 1.2), by fixing factor loadings to be equal across time, which decreased model fit according to chi-square difference testing (*p* < 0.0017), although not the AFI. Standardised factor loadings are shown in Table 3.

#### Hypothesis 2: invariance across sex

To test measurement invariance by sex, we assessed increasingly constrained models separately at each age, following similar procedures to hypothesis 1 testing. The baseline model provided good fit at all ages (Table 2; Models 2.1a-2.1e). Comparing the metric to configural model revealed no evidence of decrease in model fit at ages 7, 13 and 25, but decrease in model fit occurred at ages 10 (ΔRMSEA > 0.015,) and 15 (ΔRMSEA > 0.015 and *p* < 0.0017; Table 2, Models 2.2b and 2.2d). For the ages showing evidence of metric invariance, we tested scalar invariance, which indicated no fit decrease at ages 7 and 13, but did at age 25 (ΔRMSEA > 0.01; Table 2, Model 2.3e). Lastly, we assessed residual invariance for ages 7 and 13, suggesting no model fit decrease at age 13, but did at age 7 (Table 2; Models 2.4a, 2.4c). Standardised factor loadings and thresholds for the invariant ages are presented in Table 3 and Supplementary Table 5, respectively.

**Table 2:** Measurement invariance tests across age and sex (Parent-Rated DAWBA-ODD irritability items)

Hypothesis	Model	Age	Free Parameters	CFI	RMSEA	SRMR	VS	Δ Parameters	ΔCFI	ΔRMSEA	ΔSRMR	DIFFTEST Chi-Square	DIFFTEST *P*-Value	Decision
1: Age	1.1.Configural	7-25y	55	0.994	0.028	0.034								Accept
	1.2.Metric		47	0.993	0.028	0.034	1.1	-8	-0.001	0	0	72.765	< 0.0001	Reject
2: Sex	2.1.Configural	a.7y	18	1	0	0								Accept
		b.10y	18	1	0	0								Accept
		c.13y	18	1	0	0								Accept
		d.15y	18	1	0	0								Accept
		e.25y	18	1	0	0								Accept
	2.2.Metric	a.7y	16	1	0	0.001	2.1a	-2	0	0	0.001	0.459	0.7948	Accept
		b.10y	16	1	0.03	0.005	2.1b	-2	0	0.03	0.005	8.643	0.0133	Reject
		c.13y	16	1	0.006	0.002	2.1c	-2	0	0.006	0.002	2.270	0.3215	Accept
		d.15y	16	1	0.047	0.005	2.1d	-2	0	0.047	0.005	12.485	0.0019	Reject
		e.25y	16	1	0	0.001	2.1e	-2	0	0	0.001	0.753	0.6863	Accept
	2.3.Scalar	a.7y	14	1	0	0.001	2.2a	-2	0	0	0	0.124	0.9398	Accept
		b.10y												N/A
		c.13y	14	1	0	0.002	2.2c	-2	0	-0.006	0	0.492	0.7820	Accept
		d.15y												N/A
		e.25y	14	1	0.016	0.003	2.2e	-2	0	0.016	0.002	5.186	0.0748	Reject
	2.4 Strict	a.7y	11	1	0.018	0.005	2.3a	-3	0	0.018	0.004	14.817	0.0020	Reject
		b.10y												N/A
		c.13y	11	1	0.009	0.004	2.3c	-3	0	0.004	0.002	6.251	0.1000	Accept
		d.15y												N/A
		e.25y												N/A

**Table 3:** Standardised (StdYX) DAWBA-ODD irritability items’ loadings by age and sex (Parent-Rated)

Age	DAWBA-ODD Irritability Items	Loadings by Age	Loadings by Sex (Males)	Loadings by Sex (Females)
7 years	Severe Temper Tantrums	0.878	0.851	0.805
	Touchy and Easily Annoyed	0.907	0.922	0.915
	Angry and Resentful	0.952	0.960	0.960
10 years	Severe Temper Tantrums	0.908	0.869	0.846
	Touchy and Easily Annoyed	0.920	0.944	0.927
	Angry and Resentful	0.967	0.974	0.967
13 years	Severe Temper Tantrums	0.915	0.887	0.880
	Touchy and Easily Annoyed	0.935	0.949	0.945
	Angry and Resentful	0.972	0.974	0.972
15 years	Severe Temper Tantrums	0.925	0.899	0.922
	Touchy and Easily Annoyed	0.943	0.935	0.961
	Angry and Resentful	0.963	0.969	0.962
25 years	Severe Temper Tantrums	0.948	0.948	0.917
	Touchy and Easily Annoyed	0.932	0.947	0.939
	Angry and Resentful	0.969	0.965	0.971

DAWBA-ODD: Development and Well-Being Assessment-Oppositional Defiant Disorder. Factor loadings presented for the best-fitting models: age-invariant are from the configural model; sex-invariant loadings are from the configural (10, 15 years), metric (25 years), scalar (7 years) and strict (13 years) models (Table 2)

#### Hypothesis 3: invariance across informants

To test our third hypothesis, we first assessed a baseline invariance model separately by sex, fitting two correlated single latent factors (parent-rated and self-rated DAWBA-ODD irritability items), with free loadings and thresholds across rater. These models fitted well for both sexes (Table 4; Models 3.1.1a, 3.1.1b). Therefore, we tested metric invariance by fixing factor loadings to be equal across informants, which did not show evidence of model fit decrease for either sex (Table 4; Models 3.1.2a, 3.1.2b). Then, we tested scalar invariance by fixing the factor thresholds to be equal across raters, also showing no evidence of model fit decrease (Table 4; Models 3.1.3a, 3.1.3b). Lastly, we fixed residual variances to also be equal across raters, again showing no evidence of model fit decrease for either sex (Table 4, Models 3.1.4a, 3.1.4b). Standardised factor loadings are displayed in Table 5, and thresholds in Supplementary Table 5.

### ARI

As secondary analyses, we investigated measurement invariance across informants by sex in the ARI, following similar processes to the DAWBA-ODD irritability items informant hypothesis testing. The configural model showed good fit (Table 4; Models 3.2.1a, 3.2.1b), so we tested metric invariance. This resulted in worse fit for males (chi-square difference testing: *p* < 0.0017) but not females (Table 4; Models 3.2.2a, 3.2.2b). We then tested scalar invariance for the female model, by fixing item thresholds to be equal across informants, which did not show evidence of model fit decrease (Table 4; Model 3.2.3b). Lastly, we assessed residual invariance for the female ARI model, indicating no model fit decrease (Table 4, Model 3.2.4b). Standardised factor loadings are presented in Table 5, and for females, thresholds in Supplementary Table 5.

**Table 4:** Measurement invariance tests across informant by sex (Age 25)

Hypothesis	Model	Sex	Free Parameters	CFI	RMSEA	SRMR	VS	Δ Parameters	ΔCFI	ΔRMSEA	ΔSRMR	DIFFTEST Chi-Square	DIFFTEST *P*-Value	Decision
3.1: Informant (DAWBA-ODD Irritability)	1.Configural	a.Male	19	0.998	0.033	0.049								Accept
	2.Metric		17	0.998	0.031	0.049	3.1/1a	-2	0	-0.002	0	3.029	0.2199	Accept
	3.Scalar		15	0.998	0.029	0.050	3.1/2a	-2	0	-0.002	0.001	3.379	0.1846	Accept
	4. Residual		12	0.997	0.027	0.050	3.1/3a	-3	-0.001	-0.002	0	5.778	0.1229	Accept
	1.Configural	b.Female	19	1	0	0.02								Accept
	2.Metric		17	1	0.009	0.02	3.1/1b	-2	0	0.009	0	6.924	0.0314	Accept
	3.Scalar		15	1	0.006	0.021	3.1/2b	-2	0	-0.003	0.001	0.472	0.7898	Accept
	4. Residual		12	1	0.009	0.022	3.1/3b	-3	0	0.003	0.001	6.033	0.1100	Accept
3.2: Informant (ARI)	1.Configural	a.Male	37	0.996	0.027	0.051								Accept
	2.Metric		32	0.996	0.029	0.052	3.2/1a	-5	0	0.002	0.001	39.541	< 0.0001	Reject
	1.Configural	b.Female	37	0.995	0.032	0.034								Accept
	2.Metric		32	0.995	0.031	0.034	3.2/1b	-5	0	-0.001	0	12.605	0.274	Accept
	3.Scalar		27	0.995	0.030	0.034	3.2/2b	-5	0	-0.001	0	14.571	0.0124	Accept
	4. Residual		21	0.995	0.028	0.035	3.2/3b	-6	0	-0.002	0.001	11.316	0.0791	Accept

DAWBA-ODD: Development and Well-Being Assessment-Oppositional Defiant Disorder; ARI: Affective Reactivity Index

**Table 5:** Standardised (StdYX) DAWBA-ODD irritability items and ARI loadings across informant by sex (Age 25)

Measures	Items	Parent-Rated Loading: Male	Self-Rated Loading: Male	Parent-Rated Loading: Female	Self-Rated Loading: Female
DAWBA-ODD	Severe Temper Tantrums	0.953	0.891	0.937	0.877
Irritability Items	Touchy and Easily Annoyed	0.946	0.875	0.949	0.898
	Angry and Resentful	0.975	0.938	0.963	0.926
ARI	Easily Annoyed by Others	0.868	0.802	0.837	0.794
	Often loses temper	0.971	0.923	0.949	0.931
	Stays angry for a long time	0.903	0.814	0.899	0.830
	Angry most of the time	0.947	0.942	0.947	0.950
	Gets angry frequently	0.952	0.940	0.973	0.959
	Loses temper easily	0.956	0.875	0.942	0.906

DAWBA-ODD: Development and Well-Being Assessment-Oppositional Defiant Disorder; ARI: Affective Reactivity Index. Factor loadings presented for the best fitting models: residual invariance for the DAWBA-ODD and female ARI; configural invariance for the male ARI (Table 4)

### Sensitivity analyses using a different reference Indicator

Using the second item (DAWBA: “touchy and easily annoyed”; ARI: “often loses temper”) as the reference indicator instead of the first item (DAWBA: “severe temper tantrums”; ARI: “easily annoyed by others”) revealed a similar pattern of results to our primary analyses, with the exception of that the sex invariance tests at age 25 suggested configural, rather than metric, invariance (Supplementary Tables 8 and 9). More information is presented in the Supplementary Materials.

### Partial invariance

For the models where full invariance did not hold, we tested partial invariance; overall, these tests provided limited evidence of invariance (see Supplementary Materials). For age invariance, we found evidence of non-invariance across all items. For sex invariance at age 15, although the item “severe temper tantrums” showed partial scalar invariance alongside either (but not both) the items “touchy and easily annoyed” and “angry and resentful”, overall partial invariance was not supported, as the majority of items (“touchy and easily annoyed” and “angry and resentful”) were non-invariant. Finally for the ARI informant models in males, none of the items were found to be invariant (Supplementary Table 7).

## Discussion

This study primarily investigated measurement invariance in irritability across age, sex, and informant using the DAWBA-ODD irritability items; secondary analyses assessed rater invariance by sex in the ARI. Using the three DAWBA-ODD irritability items, we did not find evidence of even weak (metric) invariance by age (7–25 years), with differing invariance patterns found by sex across these different ages. We observed evidence of strict informant invariance (age 25) for the DAWBA-ODD irritability items across sexes, and in females, but not males, for the ARI.

Overall, our models did not support measurement invariance for ages 7–25 years. Inconsistent with hypothesis 1, the three DAWBA-ODD items did not load on one irritability factor similarly across age. This suggests that it is likely inappropriate to interpret both the individual DAWBA-ODD irritability items and their respective means similarly across age. Given that normative levels of irritability can vary developmentally [2, 16], we had expected that endorsing temper tantrum-related items in adolescence or adulthood would indicate differing levels of irritability compared to childhood. Our results are consistent with this, indicating that the extent to which individual items relate to the irritability factor, also varies developmentally, contrasting with previous research supporting strong/partial-strong invariance in irritability across more limited age ranges [21, 22]. Our findings therefore advise against using the parent-rated DAWBA-ODD irritability items to measure developmental differences in irritability across childhood, adolescence and young adulthood; however, it is unclear if this would also apply to other irritability measures or self-reports.

Our sex-invariance findings differed by age-group. For age 13, evidence of strict invariance was found, suggesting that at this age, individual irritability items’ mean scores are equivalent across sex and could therefore be used to assess sex differences; at age 7, strong invariance was suggested, indicating that factor means (but not total scores) are comparable. At age 25, only evidence of weak invariance was found, suggesting that the individual DAWBA-ODD irritability items likely tapped irritability similarly across sexes, but they were endorsed at different irritability levels in females than males; this suggests that mean comparisons may be invalid. Finally, at ages 10 and 15, our models did not support even weak invariance, arguing that the extent that different DAWBA-ODD irritability items tap irritability varies by sex at these ages. Thus, while the results at ages 10 and 15 years are consistent with our second hypothesis, at ages 7, 13 and 25, the observed invariance levels were stronger than expected. Item-level investigation suggested that at age 15 years the more behavioural DAWBA-ODD irritability item (“severe temper tantrums”) was the most invariant across sexes, with some evidence that the more emotional ones (“touchy and easily annoyed” and “angry and resentful”) were differentially indicative of irritability for females and males. Although in adolescence specifically, emotional and behavioural manifestations of irritability have been linked more strongly with female and male irritability respectively [28], our results suggest sex-specific manifestations may be more nuanced than this. Overall, our findings advise caution when investigating sex differences in irritability using the DAWBA-ODD irritability items, as irritability may not be captured equivalently across sexes.

Finally, we found evidence of strict invariance across informants by sex at age 25, suggesting that thresholds for endorsing the DAWBA-ODD irritability items may be equivalent for parent- and self-raters at this age, and therefore, individual items’ mean scores, comparable. These findings were inconsistent with our third hypothesis: we had expected items to be endorsed at different irritability levels in parent- compared to self-reports, considering that some irritability symptoms may be difficult for informants to observe [23], particularly for females, who typically display more emotional/mood-like symptomatology [27, 28]. An explanation for observing stronger invariance than expected could be that, by age 25, individuals possess the emotional maturity to reflect and report their own experience equivalently to parents. Alternatively, it might be because the DAWBA-ODD irritability items are from the ODD section, therefore assessing a more behavioural irritability aspect (e.g., temper tantrums), which parents can observe similarly with self-raters. Research in adults often relies on self-reports; however, our findings suggest that for irritability, the DAWBA-ODD irritability items may be equivalent for parent- and self-raters and hence, parent-reports could serve as a good alternative to prevent informant change across age, or minimise biases from relying exclusively on self-reports [57].

Interestingly, our secondary analyses using the ARI, unlike the DAWBA-ODD irritability items, supported strict invariance only for females (but not even weak invariance for males), suggesting that the individual ARI items may capture irritability equally across informants in females, but not in males. One explanation could be that the ARI taps emotional/affective symptoms of irritability, beyond the DAWBA-ODD conceptualisation, which parent may rate more similarly to self-reports in females than males. However, our partial invariance assessment suggested that all ARI items were non-invariant for males, providing no clear evidence of differential invariance pattern across affective vs. behavioural symptoms. Another distinction between the measures could be that the ARI assesses the threshold, frequency and duration of symptoms [11], while the DAWBA-ODD irritability does not differentiate among them: these aspects may be harder to rate for parents compared to young people themselves. Sex differences in informant (non-)invariance could be due to more open communication between parent-daughter than parent-son dyads [58]. Generally, prior research suggests that informant discrepancies in ARI are more prominent in earlier developmental periods [59], and weak rater invariance across sexes has been supported in two independent samples (mean ages 12.7 and 10.2 years) using the ARI [29]. Our findings extend this work, proposing that individual ARI items may be interpreted consistently between parents and youth in females, but not males, at age 25.

This study is the first to our knowledge exploring measurement invariance in irritability across age, sex, and informant, utilising a large longitudinal sample with the same measure and informant from childhood to adulthood. Irritability was also assessed using a second highly validated measure (ARI). Another key strength was the comprehensive assessment of measurement invariance, including testing for strict invariance, often omitted in studies using ordinal categorical items, but needed for full factorial invariance to ensure that differences in observed item responses reflect true latent construct differences for categorical/ordinal items [60]. However, limitations should be acknowledged. Measurement invariance testing is grounded on fundamentally untestable assumptions, for instance assuming a truly invariant reference indicator to accurately facilitate factor-scaling; therefore, our findings cannot and do not imply absolute conclusions. On that note, using ordinal indicators for strict invariance testing means that residual variances do not carry the same interpretation as in linear models, as they reflect threshold and scaling parameters; constraining these may impact model identification/interpretability, thus necessitating cautious interpretation. We further acknowledge that our model selection criteria, based on both approximate fit indices and Mplus difference testing, although display increased sensitivity to detecting invariance violations, may have led to suggesting trivial model fit misspecifications as statistically significant, leading to rejection of invariant models. We attempted to compensate for this by introducing Bonferroni-corrected significance levels for multiple testing, however the practical effect size of such violation warrants further investigation in future studies. The longitudinal design has also led to high attrition rates, which previous ALSPAC work suggests are likely non-random; individuals with lower education [61], increased familial adversity [36, 62] male sex [35] and higher behavioural difficulties [62] are more likely to dropout. Additionally, the DAWBA measures irritability in the context of ODD; irritability has indeed been validated as a separate ODD-dimension [38] and is used as an ODD-specifier [63, 64]. These items are inherently ODD-specific, so age and sex invariance findings may not generalise to other irritability conceptualisations, like disruptive mood dysregulation disorder (DMDD) [63]; further research looking at DMDD-like irritability across age and sex, is needed. Moreover, employing a community sample also means that findings may not generalise to clinical populations, while the ethnically non-diverse sample could also limit generalisability in diverse populations: further research in clinical and more diverse samples is needed. Future studies at additional age-groups would be beneficial too: first, investigating rater-invariance at earlier ages, as self-reports in our sample were only available in adulthood, and second, exploring invariance spanning toddlerhood and early childhood, given these periods’ developmental importance in irritability.

Findings from this study have potential implications for both clinicians and researchers. First, our results are consistent with the three DAWBA-ODD irritability items not measuring the same irritability concept similarly over time, therefore suggesting that this measure may not be compatible for investigating and monitoring developmental differences and symptom progress. Likewise, it may not be suitable for comparing mean-level sex differences; therefore, different severity cut-off points may be needed across ages and sexes. Finally, our results suggest that parent- and self-reports at age 25 can be used to measure irritability equivalently using the DAWBA-ODD irritability items, and for females specifically, the ARI; the latter, however, may not be appropriate for mean score comparisons in males.

Concluding, our study explored measurement invariance in irritability measures across age, sex, and informant, in a large longitudinal cohort. We found evidence of strict measurement invariance across informant (parent/self) at age 25 for the DAWBA-ODD irritability items, suggesting that in young adulthood, mean irritability levels are comparable across raters. Conversely, we found evidence of non-invariance by age, and mixed results for sex invariance, indicating that the DAWBA-ODD irritability items may capture irritability differently across development and across sexes. Our secondary analyses indicate strict informant invariance for females, but non-invariance for males, proposing that the ARI likely taps irritability differently by self- and parent-reports in males and suggesting the importance of considering sex differences when assessing informant invariance. Overall, our findings could suggest variability in irritability measurement across age, sex, and informant. This highlights the heterogenous nature of irritability, indicating that irritability may be interpreted differently across groups, even when the same measure is used.

## Electronic supplementary material

Below is the link to the electronic supplementary material.

Supplementary Material 1

## Data Availability

Data access for the Avon Longitudinal Study of Parents and Children is through a system of managed open access.

## Code availability

Available upon publication (https://github.com/KaterinaBek/measurement_invariance_mplus).
