# Supplementary material for: Measuring irritability across childhood, adolescence, and young adulthood: an investigation of measurement invariance by age, sex, and informant
Source: Eur Child Adolesc Psychiatry. 2025 Jul 12;35(1):121–32. doi: 10.1007/s00787-025-02817-3 (PMC12916536; doi:10.1007/s00787-025-02817-3)
Supplement: Supplementary file 1 — Supplementary Material 1 [file 787_2025_2817_MOESM1_ESM.docx]

**Measuring Irritability Across Childhood, Adolescence, and Young Adulthood: An Investigation of Measurement Invariance by Age, Sex, and Informant**

Aikaterini Bekiropoulou, Olga Eyre, Jon Heron, Anita Thapar_,_ Lucy Riglin

**Supplementary Materials**

**Supplementary Table 1.** Sample Demographic Characteristics (N=10,167)

| Demographic | Frequency (N) | Percentage (%) |
| --- | --- | --- |
| Sex |  |  |
| Male | 5056 | 49.73% |
| Female | 5100 | 50.16% |
| Missing | 11 | 0.11% |
| Ethnicity |  |  |
| White | 8661 | 85.19% |
| Non-white | 392 | 3.86% |
| Missing | 1114 | 10.96% |
| Mother’s social class by occupation |  |  |
| Professional & skilled (non-manual) | 6341 | 62.37% |
| Skilled (manual) & lower | 1843 | 18.13% |
| Missing | 1983 | 19.50% |
| Father’s social class by occupation |  |  |
| Professional & skilled (non-manual) | 4081 | 40.14% |
| Skilled (manual) & lower | 3083 | 30.33% |
| Missing | 3003 | 29.54% |
| Mother’s highest educational qualification |  |  |
| A level & higher | 3678 | 36.17% |
| O level & lower | 5161 | 54.56% |
| Missing | 1328 | 9.27%% |
| Father’s highest educational qualification |  |  |
| A level & higher | 4298 | 42.28% |
| O level & lower | 4638 | 45.62% |
| Missing | 1231 | 12.11% |
| Home ownership status |  |  |
| Owned | 7183 | 70.65% |
| Other | 1662 | 16.34% |
| Missing | 1322 | 13% |

**Supplementary Table 2.** Data Summary by Instrument

| Instrument | Age | Informant | Invariance Comparison |
| --- | --- | --- | --- |
| DAWBA-ODD Irritability Items | 7 years, 10 years, 13 years, 15 years, 25 years | Parent: 7-25 years  Self: 25 years | Parent: Age, Sex, Informant  Self: Sex, Informant |
| ARI | 25 years | Parent & Self | Sex, Informant |

DAWBA – ODD: Development and Well-Being Assessment – Oppositional Defiant Disorder;
ARI: Affective Reactivity Index

**Supplementary Table 3.** Parameter Fixing and Calculation Across Age and Sex

| Model | Factor loadings | Thresholds | Scale Factors/ Residual Variance | Factors Means | Factor Variances | Factors Covariance | Free Parameters |
| --- | --- | --- | --- | --- | --- | --- | --- |
| H1: Age |  |  |  |  |  |  |  |
| Configural | Free across factors  (first fixed at 1) [10] | Free across factors [30] | Fixed at 1 in all factors [0] | Fixed at 0 in all factors [0] | Free across factors [5] | [10] | 55 |
| Metric | Equal across factors (first fixed at 1) [2] | First item thresholds & first threshold for subsequent items equal across factors; second free [14] | Fixed at 1 in the first factor; free in the rest [12] | Fixed at 0 in the first factor; free in the rest [4] | Free across factors [5] | [10] | 47 |
| Partial Metric | Equal across factors (first fixed at 1), non-invariant free [6] | First item thresholds & first threshold of invariant item equal across factors; non-invariant thresholds and second threshold of invariant item free [18] | Fixed at 1 in all factors [0]* | Fixed at 0 in the first factor; free in the rest [4] | Free across factors [5] | [10] | 43 |
| Scalar | Equal across factors (first loading fixed  at 1) [2] | Equal across factors [6] | Fixed at 1 in the first factor; free in the rest [12] | Fixed at 0 in the first factor; free in the rest [4] | Free across factors [5] | [10] | 39 |
| H2: Sex |  |  |  |  |  |  |  |
| Configural | Free across groups  (first fixed at 1) [4] | Free across groups [12] | Fixed at 1 in both groups [0] | Fixed at 0 in both groups [0] | Free across groups [2] |  | 18 |
| Metric | Equal across groups (first fixed at 1) [2] | First item thresholds & first threshold for subsequent items equal across groups; second free [8] | Fixed at 1 in the first group; free in the second [3] | Fixed at 0 in the first group; free in the second [1] | Free across groups [2] |  | 16 |
| Partial Metric | First fixed at 1, invariant equal across groups, non-invariant free [3] | First item thresholds & first threshold of invariant item equal across groups, non-invariant thresholds and second threshold of invariant item free [9] | Free in invariant items across groups; fixed at 1 in the non-invariant [2] | Fixed at 0 in the first group; free in the second [1] | Free across groups [2] |  | 17 |
| Scalar | Equal across groups (first fixed at 1) [2] | Equal across groups [6] | Fixed at 1 in the first group; free in the second [3] | Fixed at 0 in the first group; free in the second [1] | Free across groups [2] |  | 14 |
| Partial Scalar | Equal across groups (first fixed at 1) [2] | Invariant items’ thresholds equal across groups, non-invariant’s free [8] | Free across groups in invariant; non-invariant fixed at 1[2] | Fixed at 0 in the first group; free in the second [1] | Free across groups [2] |  | 15 |
| Residual | Equal across groups (first fixed at 1) [2] | Equal across groups [6] | Fixed at 1 in both groups [0] | Fixed at 0 in the first group; free in the second [1] | Free across groups [2] |  | 11 |
| Partial Residual | Equal across groups (first fixed at 1) [2] | Equal across groups [6] | Free across groups in invariant items; non-invariant fixed at 1[2] | Fixed at 0 in the first group; free in the second [1] | Free across groups [2] |  | 13 |

H1: Hypothesis 1, H2: Hypothesis 2 | *for identification

**Supplementary Table 4.** Parameter Fixing and Calculation Across Informants by Sex

| Model | Factor loadings | Thresholds | Scale Factors/ Residual Variance | Factors Means | Factor Variances | Factors Covariance | Free Parameters |
| --- | --- | --- | --- | --- | --- | --- | --- |
| H3: Rater (DAWBA) |  |  |  |  |  |  |  |
| Configural | Free across factors  (first fixed at 1) [4] | Free across factors [12] | Fixed at 1 in both factors [0] | Fixed at 0 in both factors [0] | Free across factors [2] | [1] | 19 |
| Metric | Equal across factors (first fixed at 1) [2] | First item thresholds & first threshold for subsequent items equal across factors; second free [8] | Fixed at 1 in the first factor; free in the second [3] | Fixed at 0 in the first factors; free in the second [1] | Free across factors [2] | [1] | 17 |
| Scalar | Equal across factors (first fixed at 1) [2] | Equal across factors [6] | Fixed at 1 in the first factor; free in the second [3] | Fixed at 0 in the first factors; free in the second [1] | Free across factors [2] | [1] | 15 |
| Residual | Equal across factors (first fixed at 1) [2] | Equal across factors [6] | Fixed at 1 in both factors [0] | Fixed at 0 in the first factors; free in the second [1] | Free across factors [2] | [1] | 12 |
| H3: Rater (ARI) |  |  |  |  |  |  |  |
| Configural | Free across factors  (first fixed at 1)[10] | Free across factors [24] | Fixed at 1 in both factors [0] | Fixed at 0 in both factors [0] | Free across factors [2] | [1] | 37 |
| Metric | Equal across factors (first fixed at 1) [5] | First item thresholds & first threshold for subsequent items equal across factors; second free [17] | Fixed at 1 in the first factor; free in the second [6] | Fixed at 0 in the first factor; free in the second [1] | Free across factor [2] | [1] | 32 |
| Partial Metric  (one indicator) | First item fixed at 1, invariant equal, non-invariant free [6] | First item thresholds & first threshold of invariant items equal across factors; non-invariant thresholds and second threshold of invariant items free [18] | Fixed at 1 in both factors [0]* | Fixed at 0 in the first factor; free in the second [1] | Free across factors [2] | [1] | 28 |
| Partial Metric  (three indicators) | First item fixed at 1, invariant equal, non-invariant free [8] | First item thresholds & first threshold of invariant items equal across factors; non-invariant thresholds and second threshold of invariant items free [20] | Fixed at 1 in both factors [0]* | Fixed at 0 in the first factor; free in the second [1] | Free across factors [2] | [1] | 32 |
| Scalar | Equal across factors (first fixed at 1) [5] | Equal across factors [12] | Fixed at 1 in the first factor; free in the second [6] | Fixed at 0 in the first factor; free in the second [1] | Free across factor [2] | [1] | 27 |
| Residual | Equal across factors (first fixed at 1) [5] | Equal across factors [12] | Fixed at 1 in both factors [0] | Fixed at 0 in the first factor; free in the second [1] | Free across factor [2] | [1] | 21 |

DAWBA: Development & Well-Being Assessment (Irritability Items); ARI: Affective Reactivity Index, H3: Hypothesis 3|*for identification

**Supplementary Table 5.** Standardised (StdYX) DAWBA-ODD Irritability Items’ Factor Thresholds Across Age by Sex (Invariant)

| Age | DAWBA-ODD Irritability Items/Threshold | Thresholds: Males | Thresholds: Females |
| --- | --- | --- | --- |
| 7 years | Severe Temper Tantrums (1) | 1.249 | 1.491 |
|  | Severe Temper Tantrums (2) | 1.940 | 2.316 |
|  | Touchy & Easily Annoyed(1) | 0.796 | 0.998 |
|  | Touchy & Easily Annoyed(2) | 1.808 | 2.266 |
|  | Angry & Resentful (1) | 1.039 | 1.312 |
|  | Angry & Resentful (2) | 1.828 | 2.309 |
| 10 years | Severe Temper Tantrums (1) |  |  |
|  | Severe Temper Tantrums (2) |  |  |
|  | Touchy & Easily Annoyed(1) |  |  |
|  | Touchy & Easily Annoyed(2) |  |  |
|  | Angry & Resentful (1) |  |  |
|  | Angry & Resentful (2) |  |  |
| 13 years | Severe Temper Tantrums (1) | 1.452 | 1.498 |
|  | Severe Temper Tantrums (2) | 2.113 | 2.179 |
|  | Touchy & Easily Annoyed(1) | 0.894 | 0.926 |
|  | Touchy & Easily Annoyed(2) | 1.937 | 2.007 |
|  | Angry & Resentful (1) | 1.149 | 1.192 |
|  | Angry & Resentful (2) | 1.961 | 2.035 |
| 15 years | Severe Temper Tantrums (1) |  |  |
|  | Severe Temper Tantrums (2) |  |  |
|  | Touchy & Easily Annoyed(1) |  |  |
|  | Touchy & Easily Annoyed(2) |  |  |
|  | Angry & Resentful (1) |  |  |
|  | Angry & Resentful (2) |  |  |
| 25 years | Severe Temper Tantrums (1) |  |  |
|  | Severe Temper Tantrums (2) |  |  |
|  | Touchy & Easily Annoyed(1) |  |  |
|  | Touchy & Easily Annoyed(2) |  |  |
|  | Angry & Resentful (1) |  |  |
|  | Angry & Resentful (2) |  |  |

DAWBA-ODD: Development and Well-Being Assessment-Oppositional Defiant Disorder. Thresholds presented for the scalar (7 years) and residual
variance models (13 years); evidence of scalar invariance was not supported at ages 10, 15, and 25, hence these thresholds are not displayed (Table 2)

**Supplementary Table 6.** Standardised (StdYX) DAWBA-ODD Irritability and ARI Factor Thresholds Across Informant by Sex (Invariant)

| Measures | Items/Threshold | Parent-Rated  Threshold: Male | Self-Rated  Threshold: Male | Parent-Rated  Threshold: Female | Self-Rated  Threshold: Female |
| --- | --- | --- | --- | --- | --- |
| DAWBA - ODD | Severe Temper Tantrums (1) | 1.797 | 2.710 | 1.671 | 2.297 |
| Irritability | Severe Temper Tantrums (2) | 2.267 | 3.419 | 2.304 | 3.166 |
|  | Touchy and Easily Annoyed (1) | 1.188 | 1.775 | 0.977 | 1.358 |
|  | Touchy and Easily Annoyed (2) | 2.065 | 3.084 | 1.909 | 2.654 |
|  | Angry and Resentful (1) | 1.420 | 2.205 | 1.328 | 1.874 |
|  | Angry and Resentful (2 ) | 1.998 | 3.104 | 2.009 | 2.835 |
| ARI | Easily annoyed by others (1) |  |  | 0.433 | 0.490 |
|  | Easily annoyed by others (2) |  |  | 1.745 | 1.973 |
|  | Often loses temper (1) |  |  | 1.209 | 1.424 |
|  | Often loses temper (2) |  |  | 2.088 | 2.460 |
|  | Stays angry for long (1) |  |  | 1.331 | 1.527 |
|  | Stays angry for long (2) |  |  | 2.080 | 2.386 |
|  | Angry most of the time (1) |  |  | 1.857 | 2.197 |
|  | Angry most of the time (2) |  |  | 2.486 | 2.940 |
|  | Gets angry frequently (1) |  |  | 1.448 | 1.722 |
|  | Gets angry frequently (2) |  |  | 2.239 | 2.662 |
|  | Loses temper easily (1) |  |  | 1.245 | 1.458 |
|  | Loses temper easily (2) |  |  | 2.100 | 2.359 |

DAWBA-ODD: Development and Well-Being Assessment – Oppositional Defiant Disorder Section (Irritability Items); ARI: Affective Reactivity Index. Factor thresholds presented for the residual invariance models; the male ARI was found to be non-invariant, therefore its thresholds are not displayed (Table 4)

**Partial Invariance Testing**

Partial invariance was examined using modification indices and the backwards selection method. Modification indices (MI) identify which parameters (e.g., loadings, thresholds) specifically contribute most to model misfit, therefore suggesting which parameters, if freed, can lead to significant model fit improvement [1,2]. In our study, MIs were calculated for all constrained parameters and items with the highest MIs were freed iteratively, in descending order of largest misfit, while the remaining parameters were held constrained. The DIFFTEST chi-square was used for model comparison, in line with recommendations; for residual invariance, theta parameterization was used [2]. Testing of partial invariance was limited to cases where the DIFFTEST chi-square indicated non-invariance in the full invariance models, as otherwise partial invariance may be concluded based on DIFFTEST, because DIFFTEST suggested full invariance. As recommended [3], partial invariance was supported only if the majority of a measure’s items (2 for the DAWBA-ODD, 4 for the ARI) demonstrated evidence of invariance at a loading (metric), threshold (scalar) and/or residual variances (residual) level. In cases where evidence of partial invariance was not observed upon freeing the parameters of the items with the highest MI individually, exploratory adjustments were conducted, including testing multiple items with the highest MI, to assess whether this could address model misfit more effectively. Partial invariance is considered an alternative to regularisation [4] and alignment optimisation [5], adding to our study’s robustness.

*Hypothesis 1: Partial Invariance Across Age*

Modification indices suggested that the item “severe temper tantrums” had the highest impact in model misfit (MI=84.701), compared to the other two items of the DAWBA-ODD irritability. We therefore started our post-hoc partial invariance investigation by freeing this item’s loadings and thresholds, whilst keeping the other items’ parameters constrained across age. This resulted in evidence of model fit decrease (Supplementary Table 7, Model p1.2/i.a). We then freed the item with the second largest MI (“touchy and easily annoyed”; MI=35.676) and retested the model (Supplementary Table 7, Model p1.2/i.b): this also did not suggest partial metric invariance. As two items indicated non-invariance, partial invariance criteria were not met. We also assessed the last item (“angry and resentful”), but this again did not provide evidence of partial metric invariance (Supplementary Table 7).

*Hypothesis 2: Partial Invariance Across Sex*

Partial invariance was not examined at ages 10 and 25 (scalar) and 7 (residual) as the Mplus difference test suggested full scalar and residual invariance at these ages, respectively. At age 15, modification indices suggested that item contributing most significantly to model misfit was “touchy and easily annoyed” (MI=12.274 for males and 12.277 for females). We therefore first freed this item’s loadings (partial metric invariance testing), which suggested no decrease in model fit (Supplementary Table 7). We then tested partial scalar invariance, which also did not decrease model fit. Lastly, we assessed residual invariance by freeing the residual variances of the tested item; this provided evidence of model fit decrease (Supplementary Table 7), therefore this invariance level was not supported. We repeated the same procedure for the other two DAWBA-ODD irritability items (in separate models), in decreasing order of largest misfit; this provided evidence of partial invariance at a scalar level when freeing the parameters of “angry and resentful”, but not even metric when freeing the loadings of “severe temper tantrums”.

*Hypothesis 3: Partial Invariance Across Informants in males: ARI*

We examined partial metric invariance in the male ARI model, by running models with parameters freed for one item at a time; this did not provide evidence of partial metric invariance (Supplementary Table 7). We next examined freeing the loadings of the 3 items with the highest MI, to assess whether freeing multiple parameters at the same time would suggest evidence of partial metric invariance; this also provided evidence of model fit decrease.

**Bonferroni Correction**

One of our model fit evaluation criteria, for both the full and the partial invariance models, included the chi-square difference testing, conducted using the DIFFTEST option in Mplus, which is a variant of the standard method which is appropriate for the WLSMV estimator [2]. As this approach may be oversensitive to minor misspecifications in large sample sizes [6], and because of the large number of planned tests, we used Bonferroni correction when using this statistic, to control for type 1 error. The total number of tests for the full invariance models was calculated as 30: metric, scalar and residual models for (i) age invariance (3), (ii) sex invariance for 5 ages (15), (iii) informant invariance for 2 sexes (6), and (iv) ARI informant invariance x2 sexes (6). We therefore employed Bonferroni-corrected p-value of 0.0017. For the partial invariance testing, Bonferroni-corrected p-value for the 17 partial invariance models (Supplementary Tables 6, 7) was calculated as 0.0029 (p=0.05/17=0.0029).

**Supplementary Table 7. Partial Measurement Invariance Testing**

| Hypothesis | Model | Age | Item | Modification Indices^1^ | Free Parameters | VS | Δ  Parameters | DIFFTEST  Chi-Square | DIFFTEST  P-Value^3^ | Decision |
| --- | --- | --- | --- | --- | --- | --- | --- | --- | --- | --- |
| 1.Age | **1.1 Configural** | 7-25 |  |  | 55 |  |  |  |  |  |
|  | p1.2 Partial Metric (i.a) |  | Severe Temper Tantrums | 84.701 | 43 | 1.1 | -12 | 47.258 | <0.0001 | Reject |
|  | p1.2 Partial Metric (ii.a) |  | Touchy & Easily Annoyed | 35.676 | 43 | 1.1 | -12 | 72.385 | <0.0001 | Reject |
|  | p1.2 Partial Metric (iii.a) |  | Angry & Resentful | 24.788 | 43 | 1.1 | -12 | 50.225 | <0.0001 | Reject |
| 2.Sex | **2.1d Configural** |  |  |  | 18 |  |  |  |  |  |
|  | p2.2d Partial Metric (i) | 15 | Touchy & Easily Annoyed | 12.274 (male) /  12.277 (female) | 17 | 2.1d | -1 | 1.370 | 0.2419 | Accept |
|  | p2.3d Partial Scalar (i) |  |  | 8.833 (male) /  8.837 (female) | 15 | p2.2d (i) | -2 | 1.529 | 0.4656 | Accept |
|  | p2.4d Partial Residual (i) |  |  | 6.426 (male) /  4.958 (female) | 13 | p2.3d (i) | -2 | 15.297 | 0.0005 | Reject |
|  | p2.2d Partial Metric (ii) |  | Angry & Resentful | 10.773 (male) /  10.767 (female) | 17 | 2.1d | -1 | 2.762 | 0.0966 | Accept |
|  | p2.3d Partial Scalar (ii) |  |  | 7.611 (male) /  7.602 (female) | 15 | p2.2d (ii) | -2 | 0.618 | 0.7343 | Accept |
|  | p2.4d Partial Residual (ii) |  |  | 7.200 (male) /  10.447(female) | 13 | p2.3d (ii) | -2 | 14.486 | 0.0007 | Reject |
|  | p2.2d Partial Metric (iii) |  | Severe Temper Tantrums | 2.740 (male) /  2.740 (female) | 17 | 2.1d | -1 | 10.970 | 0.0009 | Reject |
| 3. ARI (male) | 3.ARI | 25 | **3.2.1 Configural (a)** |  | 37 |  |  |  |  | Reject |
|  | p3.2.2 Partial Metric (i) |  | Easily Annoyed by Others | 20.280 | 28 | 3.2.1 | -9 | 45.443 | <0.0001 | Reject |
|  | p3.2.2 Partial Metric (ii) |  | Loses Temper Easily | 14.048 | 28 | 3.2.1 | -9 | 59.800 | <0.0001 | Reject |
|  | p3.2.2 Partial Metric (iii) |  | Angry Most of the Time | 5.497 | 28 | 3.2.1 | -9 | 50.885 | <0.0001 | Reject |
|  | p3.2.2 Partial Metric (vi) |  | Often Loses Temper | 5.520 | 28 | 3.2.1 | -9 | 58.444 | <0.0001 | Reject |
|  | p3.2.2 Partial Metric (v) |  | Gets Angry Frequently | 4.584 | 28 | 3.2.1 | -9 | 53.502 | <0.0001 | Reject |
|  | p3.2.2 Partial Metric (vi) |  | Stays Angry for Long | 0.963 | 28 | 3.2.1 | -9 | 63.726 | <0.0001 | Reject |
|  | p3.2.2 Partial Metric (vii)^2^ |  | Models’ i,ii,iii items |  | 32 | 3.2.1 | -5 | 21.502 | 0.0007 | Reject |

^1^Modification indices (mi) presented are item-specific and in descending order; they reflect the loading (metric), threshold (scalar) or residual variances (residual) with
the highest MI in the corresponding full invariance model for each item | ^2^The 3 items with the highest mi also freed together in the same model; ARI: Affective Reactivity Index

**Sensitivity Analyses Using Another Reference Indicator**

In the absence of strong theoretical rationale guiding our selection of reference indicators a priori, our primary analyses adopted the standard approach the first item of each scale was initially set as the reference indicator. Recognising that a non-invariant reference indicator may result in biased models [1], we conducted sensitivity analyses, using the second item of each measure as the reference indicator, instead of the first one, which we had provisionally set for models presented in Tables 2 and 4. For the models assessing age and informant invariance using the three DAWBA-ODD irritability items (Supplementary Tables 8 and 9, respectively), the same invariance levels were concluded as those in the initial models. For the sex invariance models (Supplementary Table 8), results suggested the same level of invariance for ages 7, 10, 13 and 15, but at age 25, changing the indicator suggested configural invariance (Supplementary Table 8), while the initial models provided evidence of metric invariance (Table 2). For the ARI invariance models, the sensitivity analyses suggested the same level of invariance as the original results (Supplementary Table 9 and Table 4, respectively).

**Supplementary Table 8.** Measurement Invariance Tests Across Age and Sex (Parent-Rated DAWBA-ODD Irritability Items) / Second Item as Reference Indicator

| Hypothesis | Model | Age | Free Parameters | CFI | RMSEA | SRMR | VS | Δ Parameters | ΔCFI | ΔRMSEA | ΔSRMR | DIFFTEST  (Chi-Square) | Decision |
| --- | --- | --- | --- | --- | --- | --- | --- | --- | --- | --- | --- | --- | --- |
| 1: Age | 1.1.Configural | 7-25y | 55 | 0.994 | 0.028 | 0.034 |  |  |  |  |  |  | Accept |
|  | 1.2.Metric |  | 47 | 0.993 | 0.028 | 0.034 | 1.1 | -8 | -0.001 | 0 | 0 | 70.965  (p<0.0001) | Reject |
| 2: Sex | 2.1.Configural | a.7y | 18 | 1 | 0 | 0 |  |  |  |  |  |  | Accept |
|  |  | b.10y | 18 | 1 | 0 | 0 |  |  |  |  |  |  | Accept |
|  |  | c.13y | 18 | 1 | 0 | 0 |  |  |  |  |  |  | Accept |
|  |  | d.15y | 18 | 1 | 0 | 0 |  |  |  |  |  |  | Accept |
|  |  | e.25y | 18 | 1 | 0 | 0 |  |  |  |  |  |  | Accept |
|  | 2.2.Metric | a.7y | 16 | 1 | 0 | 0.001 | 2.1a | -2 | 0 | 0 | 0.001 | 0.427  (p=0.8078) | Accept |
|  |  | b.10y | 16 | 1 | 0.028 | 0.005 | 2.1b | -2 | 0 | 0.028 | 0.005 | 7.833  (p=0.0199) | Reject |
|  |  | c.13y | 16 | 1 | 0 | 0.002 | 2.1c | -2 | 0 | 0 | 0.002 | 1.255  (p=0.5340) | Accept |
|  |  | d.15y | 16 | 1 | 0.04 | 0.005 | 2.1d | -2 | 0 | 0.04 | 0.005 | 9.645  (p=0.0080) | Reject |
|  |  | e.25y | 16 | 1 | 0.026 | 0.003 | 2.1e | -2 | 0 | 0.026 | 0.003 | 4.900  (p=0.0863) | Reject |
|  | 2.3.Scalar | a.7y | 14 | 1 | 0 | 0.001 | 2.2a | -2 | 0 | 0 | 0 | 0.057  (p=0.9718) | Accept |
|  |  | b.10y |  |  |  |  |  |  |  |  |  |  | N/A |
|  |  | c.13y | 14 | 1 | 0 | 0.002 | 2.2c | -2 | 0 | 0 | 0 | 1.244  (p=0.5369) | Accept |
|  |  | d.15y |  |  |  |  |  |  |  |  |  |  | N/A |
|  |  | e.25y |  |  |  |  |  |  |  |  |  |  | N/A |
|  | 2.4 Residual | a.7y | 11 | 1 | 0.018 | 0.005 | 2.3a | -3 | 0 | 0.018 | 0.004 | 15.028  (p=0.0018) | Reject |
|  |  | b.10y |  |  |  |  |  |  |  |  |  |  | N/A |
|  |  | c.13y | 11 | 1 | 0.011 | 0.004 | 2.3c | -3 | 0 | 0.011 | 0.002 | 7.430  (p=0.0594) | Accept |
|  |  | d.15y |  |  |  |  |  |  |  |  |  |  | N/A |
|  |  | e.25y |  |  |  |  |  |  |  |  |  |  | N/A |

DAWBA-ODD: Development and Well-Being Assessment - Oppositional Defiant Disorder. Primary results using the first item as the reference indicator are presented in Table 2

**Supplementary Table 9**. Measurement Invariance Tests Across Informant / Second Item as Reference Indicator

| Hypothesis | Model | Sex | Free Parameters | CFI | RMSEA | SRMR | VS | Δ Parameters | ΔCFI | ΔRMSEA | ΔSRMR | DIFFTEST  Chi-Square | Decision |
| --- | --- | --- | --- | --- | --- | --- | --- | --- | --- | --- | --- | --- | --- |
| 3.1: Informant (DAWBA-ODD | 1.Configural | a.Male | 19 | 0.998 | 0.033 | 0.049 |  |  |  |  |  |  | Accept |
| Irritability Items) | 2.Metric |  | 17 | 0.998 | 0.031 | 0.049 | 3.1/1a | -2 | 0 | -0.002 | 0 | 3.515  (p=0.1724) | Accept |
|  | 3.Scalar |  | 15 | 0.998 | 0.029 | 0.050 | 3.1/2a | -2 | 0 | -0.002 | 0.001 | 2.924  (p=0.2317) | Accept |
|  | 4. Strict |  | 12 | 0.997 | 0.027 | 0.050 | 3.1/3a | -3 | -0.001 | -0.002 | 0 | 5.809  (p=0.1213) | Accept |
|  | 1.Configural | b.Female | 19 | 1 | 0 | 0.02 |  |  |  |  |  |  | Accept |
|  | 2.Metric |  | 17 | 1 | 0.008 | 0.021 | 3.2/1b | -2 | 0 | 0.008 | 0.001 | 5.957  (p=0.0509) | Accept |
|  | 3.Scalar |  | 15 | 1 | 0.006 | 0.021 | 3.2/2b | -2 | 0 | -0.002 | 0 | 0.500  (p=0.7790) | Accept |
|  | 4. Strict |  | 12 | 1 | 0.009 | 0.022 | 3.1/3b | -3 | 0 | 0.003 | 0.001 | 5.970  (p=0.1131) |  |
| 3.2: Informant (ARI) | 1.Configural | a.Male | 37 | 0.996 | 0.027 | 0.051 |  |  |  |  |  |  | Accept |
|  | 2.Metric |  | 32 | 0.996 | 0.028 | 0.052 | 3.2/1a | -5 | 0 | 0.001 | 0.001 | 32.493  (p<0.0001) | Reject |
|  | 1.Configural | b.Female | 37 | 0.995 | 0.032 | 0.034 |  |  |  |  |  |  | Accept |
|  | 2.Metric |  | 32 | 0.995 | 0.031 | 0.034 | 3.2/1b | -5 | 0 | -0.001 | 0 | 18.903  (p=0.0020) | Accept |
|  | 3.Scalar |  | 27 | 0.995 | 0.030 | 0.034 | 3.2/2b | -5 | 0 | -0.001 | 0 | 9.989  (p=0.0756) | Accept |
|  | 4. Strict |  | 21 | 0.995 | 0.028 | 0.035 | 3.2/3b | -6 | 0 | -0.002 | 0.001 | 11.314  (p=0.0791) | Accept |

DAWBA-ODD: Development and Well-Being Assessment - Oppositional Defiant Disorder; ARI: Affective Reactivity Index. Primary results using the first item as the reference indicator are presented in Table 4

**Response Rates**

Our sample’s response rates suggest that the greatest discrepancy for group sizes is less than 2:1, which is not considered severely unbalanced in this regard [7]. Information on specific number of responses per measure/item, is presented in Supplementary Table 9.

| Item | Measure | Age | Rater | Male  (By Sex/Rater) | Female  (By Sex/Rater) | Total  (By Age) |
| --- | --- | --- | --- | --- | --- | --- |
| Severe Temper Tantrums | DAWBA-ODD Irritability Items | 7 | Parent | 4,108 | 3,889 | 7,990 |
|  |  | 10 |  | 3,825 | 3,760 | 7,585 |
|  |  | 13 |  | 3,445 | 3,437 | 6,884 |
|  |  | 15 |  | 2,221 | 2,417 | 4,644 |
|  |  | 25 |  | 2,013 | 2,320 | 4,333 |
|  |  | 25 | Self | 1,353 | 2,699 | - |
| Touchy & Easily Annoyed |  | 7 | Parent | 4,110 | 3,898 | 8,008 |
|  |  | 10 |  | 3,825 | 3,758 | 7,583 |
|  |  | 13 |  | 3,460 | 3,445 | 6,905 |
|  |  | 15 |  | 2,212 | 2,421 | 4,639 |
|  |  | 25 |  | 2,013 | 2,326 | 4,339 |
|  |  | 25 | Self | 1,353 | 2,704 | - |
| Angry & Resentful |  | 7 | Parent | 4,102 | 3,888 | 7,990 |
|  |  | 10 |  | 3,820 | 3,756 | 7,576 |
|  |  | 13 |  | 3,447 | 3,438 | 6,885 |
|  |  | 15 |  | 2,212 | 2,419 | 4,637 |
|  |  | 25 |  | 2,010 | 2,321 | 4,331 |
|  |  | 25 | Self | 1,353 | 2,696 | - |
| Easily Annoyed by Others | ARI | 25 | Parent | 2,023 | 2,341 | - |
|  |  |  | Self | 1,355 | 2,702 | - |
| Often Loses Temper |  |  | Parent | 2,026 | 2,341 | - |
|  |  |  | Self | 1,357 | 2,703 | - |
| Stays Angry for a Long Time |  |  | Parent | 2,027 | 2,338 | - |
|  |  |  | Self | 1,353 | 2,702 | - |
| Angry Most of the Time |  |  | Parent | 2,023 | 2,335 | - |
|  |  |  | Self | 1,355 | 2,699 | - |
| Gets Angry Frequently |  |  | Parent | 2,026 | 2,339 | - |
|  |  |  | Self | 1,354 | 2,699 | - |
| Loses Temper Easily |  |  | Parent | 2,024 | 2,332 | - |
|  |  |  | Self | 1,354 | 2,700 | - |

**Supplementary Table 10.** Response Rates by Item, Across Measures, Ages, Sexes and Raters

DAWBA-ODD: Development and Well-Being Assessment – Oppositional Defiant Disorder Section;
ARI: Affective Reactivity Index


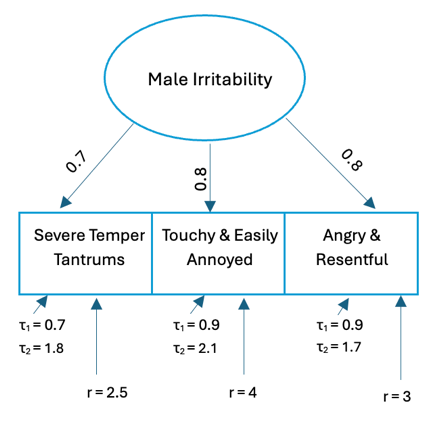
**
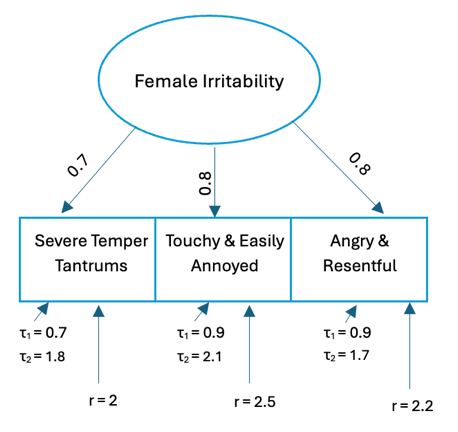
**
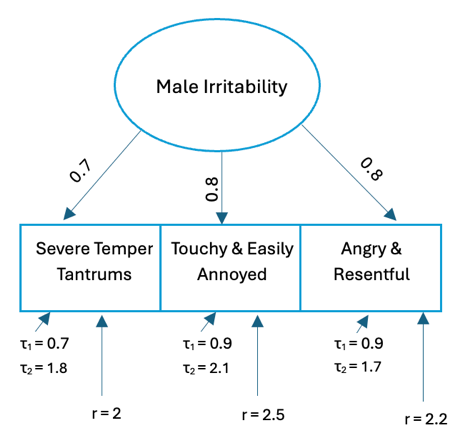
**
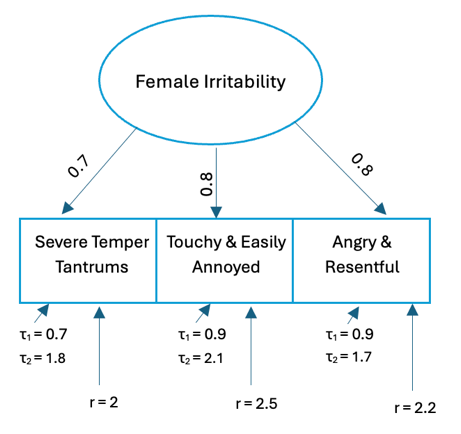
**
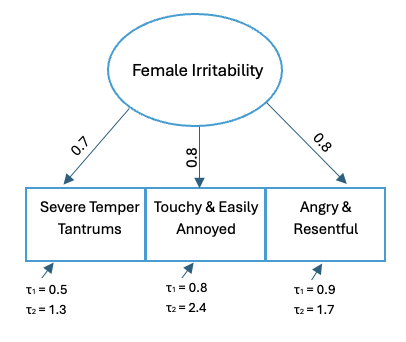

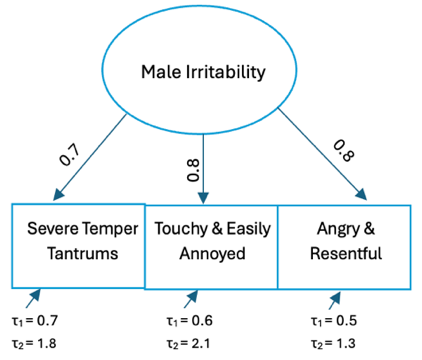

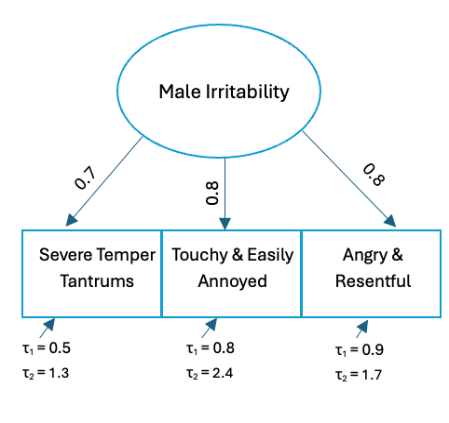

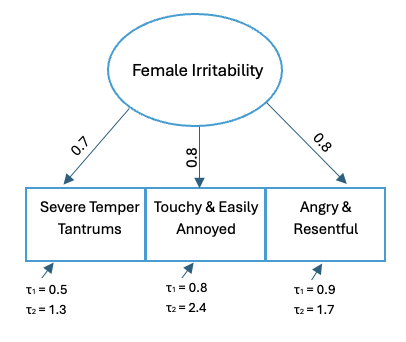

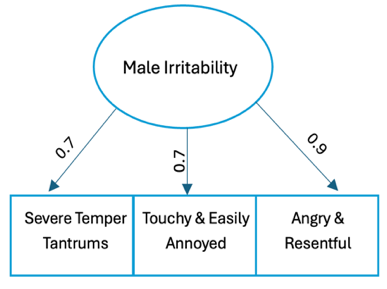

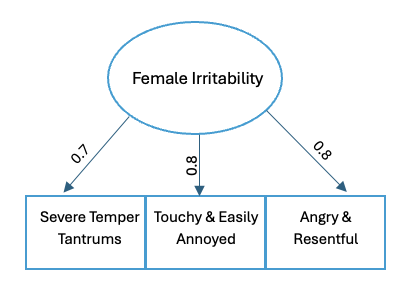

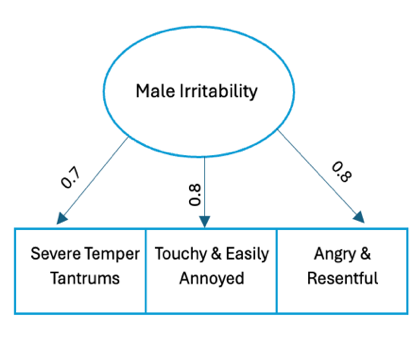

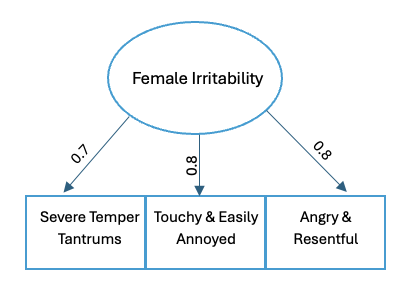

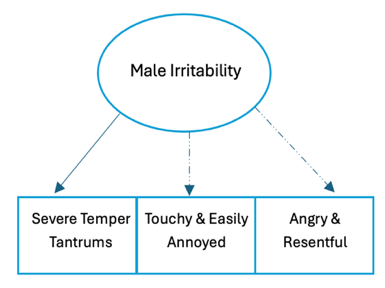

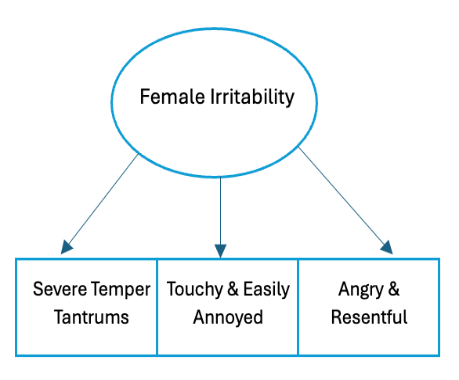

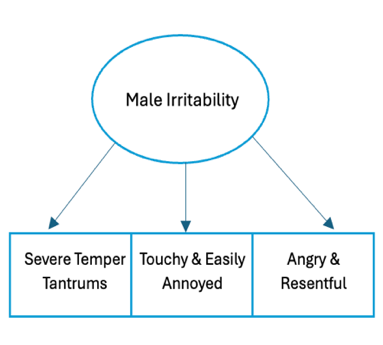

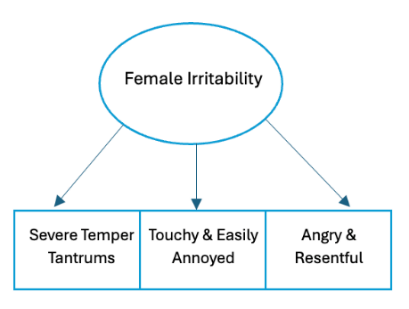
**Supplementary Figure 1.** Examples of Measurement (Non) Invariance by Sex

h) Example of Residual / “Strict” Non-Invariance

g) Example of Residual / “Strict” Invariance

d) Example of Metric / “Weak” Non-Invariance

c) Example of Metric / “Weak” Invariance

e) Example of Scalar / “Strong” Invariance

f) Example of Scalar / “Strong” Non-Invariance

a) Example of Configural Invariance

b) Example of Configural Non-Invariance

Adapted by Putnick & Bornstein [8]; τ = threshold (i.e., the level of the underlying construct at which a rater endorses an item); r = residual; (i.e., sum of item-specific variance and error variance)

**Supplementary Figure 2.** Prevalence of Irritability Items Across Age, as Measured by the DAWBA-ODD Irritability Items

1b. Touchy and Easily Annoyed

1a. Severe Temper Tantrums

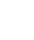

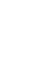

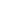


1c. Angry and Resentful

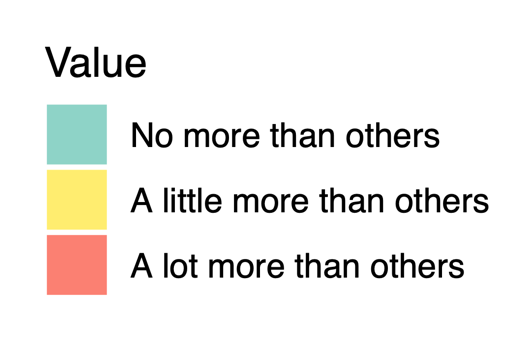


DAWBA-ODD: Development and Well-Being – Oppositional Defiant Disorder Sub-Scale

**Supplementary Figure 3.** Prevalence of Irritability Items Across Age and Sex, as Measured by the DAWBA-ODD Irritability Items

2b. Touchy and Easily Annoyed

2a. Severe Temper Tantrums

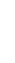


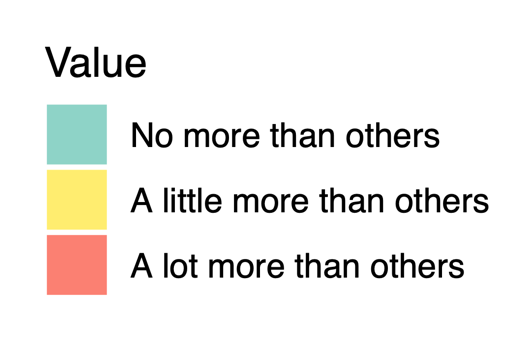


2c. Angry and Resentful

DAWBA: Development and Well-Being Assessment – Defiant Disorder Sub-Scale

**Supplementary Figure 4.** Prevalence of Irritability Items Across Informant (parent/self), as Measured by the DAWBA-ODD Irritability Items

3b. Self-Reported DAWBA Items

3a. Parent-Reported DAWBA Items


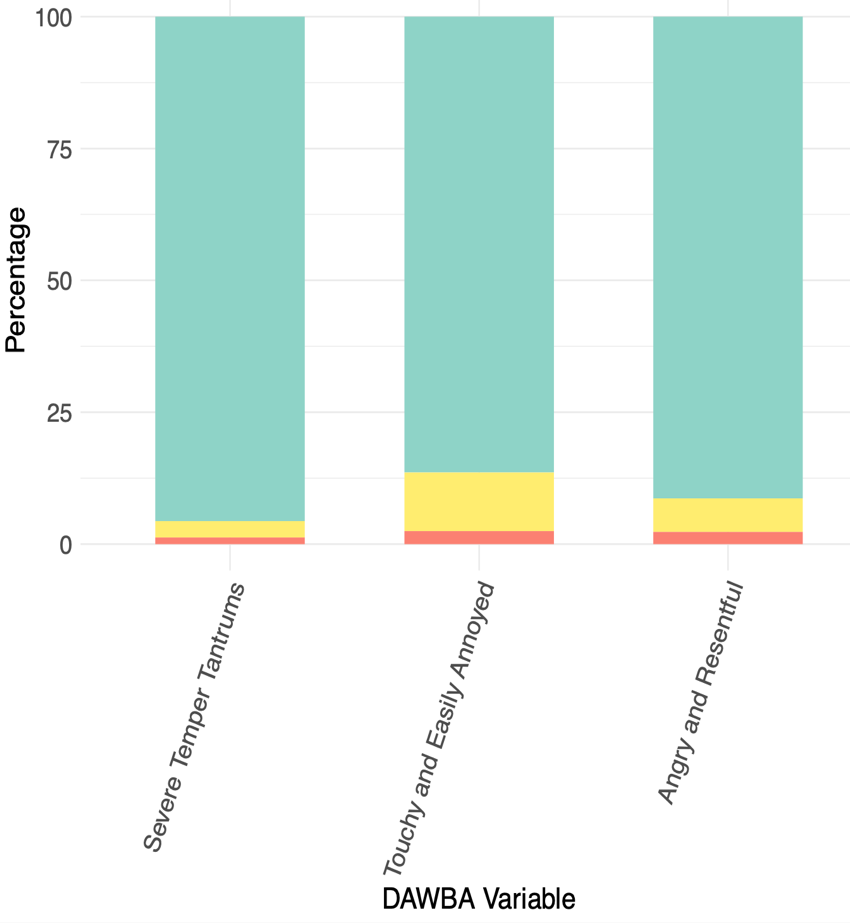


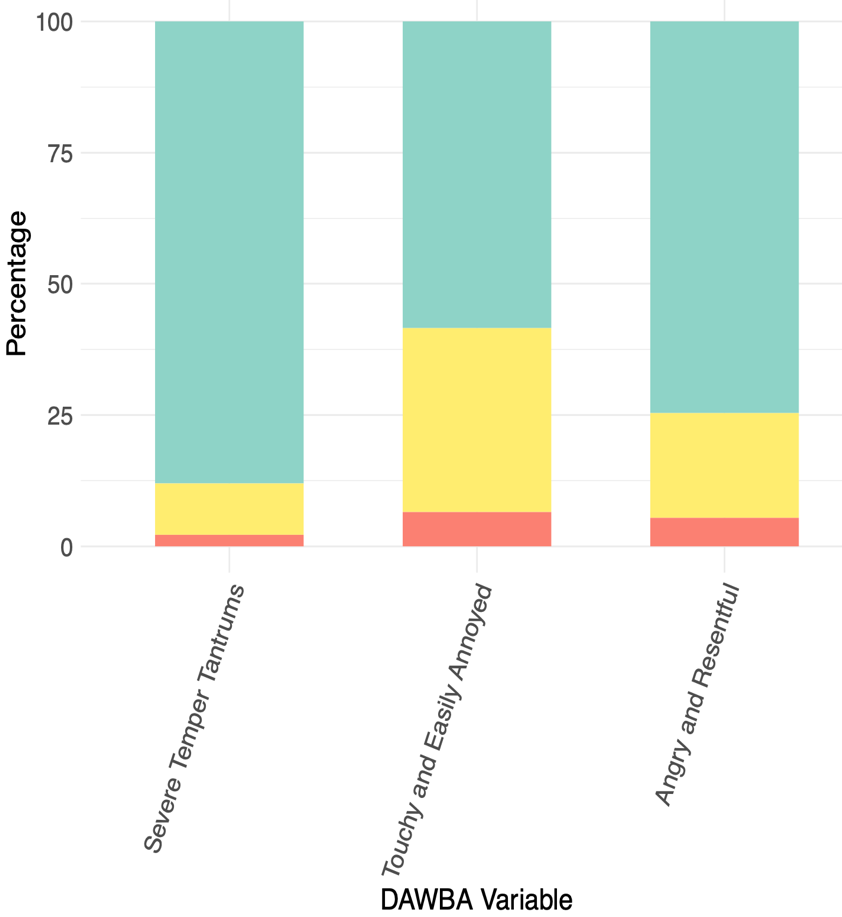


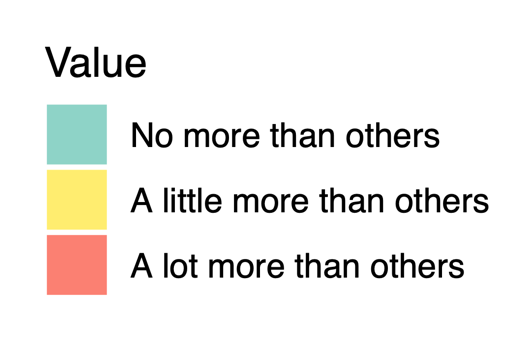


DAWBA-ODD: Development and Well-Being – Oppositional Defiant Disorder Sub-Scale. Data presented at age 25.

**Supplementary Figure 5.** Prevalence of Irritability Items Across Informant (parent/self), as Measured by
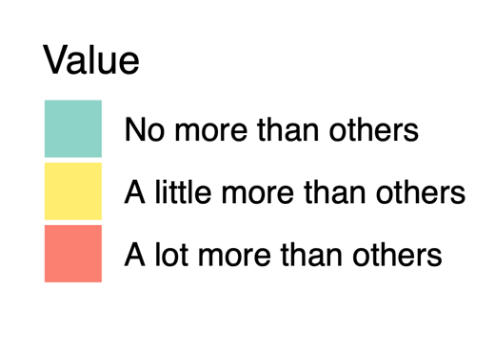
 ARI

4b. Self-Reported ARI Items

4a. Parent-Reported ARI Items

ARI: Affective Reactivity Index. Data presented at age 25.

**References**

1. Yoon M, Millsap RE. Detecting Violations of Factorial Invariance Using Data-Based Specification Searches: A Monte Carlo Study. Struct Equ Modeling. 2007 Jul 31;14(3):435–63.

2. Linda K. Muthén, Bengt O. Muthén. Mplus User’s Guide. 8th ed. Los Angeles, CA: Muthén & Muthén; 2017.

3. Vandenberg RJ, Lance CE. A Review and Synthesis of the Measurement Invariance Literature: Suggestions, Practices, and Recommendations for Organizational Research. Organ Res Methods. 2000;3(1):4–70.

4. Robitzsch A, Lüdtke O. Why Full, Partial, or Approximate Measurement Invariance Are Not a Prerequisite for Meaningful and Valid Group Comparisons. Struct Equ Modeling. 2023 Nov 2;30(6):859–70.

5. Leitgöb H, Seddig D, Asparouhov T, Behr D, Davidov E, De Roover K, et al. Measurement invariance in the social sciences: Historical development, methodological challenges, state of the art, and future perspectives. Soc Sci Res. 2023 Feb;110:102805.

6. Meade AW, Johnson EC, Braddy PW. Power and sensitivity of alternative fit indices in tests of measurement invariance. Journal of Applied Psychology. 2008 May;93(3):568–92.

7. Yoon M, Lai MHC. Testing Factorial Invariance With Unbalanced Samples. Struct Equ Modeling. 2018 Mar 4;25(2):201–13.

8. Putnick DL, Bornstein MH. Measurement invariance conventions and reporting: The state of the art and future directions for psychological research. Developmental Review. 2016 Sep;41:71–90.
